# Advancements in Titanium Dioxide Nanotube-Based Sensors for Medical Diagnostics: A Two-Decade Review

**DOI:** 10.3390/nano15131044

**Published:** 2025-07-05

**Authors:** Joydip Sengupta, Chaudhery Mustansar Hussain

**Affiliations:** 1 Department of Electronic Science, Jogesh Chandra Chaudhuri College, Kolkata 700033, India; joydipdhruba@gmail.com; 2 Department of Chemistry and Environmental Science, New Jersey Institute of Technology, Newark, NJ 07102, USA

**Keywords:** titanium dioxide nanotubes, sensing technologies, long-term operational stability, device scalability, fabrication reproducibility

## Abstract

Over the past two decades, titanium dioxide nanotubes (TiO_2_ NTs) have gained considerable attention as multifunctional materials in sensing technologies. Their large surface area, adjustable morphology, chemical stability, and photoactivity have positioned them as promising candidates for diverse sensor applications. This review presents a broad overview of the development of TiO_2_ NTs in sensing technologies for medical diagnostics over the last two decades. It further explores strategies for enhancing their sensing capabilities through structural modifications and hybridization with nanomaterials. Despite notable advancements, challenges such as device scalability, long-term operational stability, and fabrication reproducibility remain. This review outlines the evolution of TiO_2_ NT-based sensors for medical diagnostics, highlighting both foundational progress and emerging trends, while providing insights into future directions for their practical implementation across scientific and industrial domains.

## 1. Introduction

The rapid evolution of sensing technologies has revolutionized medical diagnostics [1], enabling early disease detection, real-time health monitoring, and personalized therapeutic interventions. Among the diverse nanomaterials explored for biosensing applications, TiO_2_ NTs have emerged as a highly promising platform due to their unique structural, electronic, and chemical properties [2,3,4,5]. Over the past two decades, TiO_2_ NTs have garnered significant attention for their high surface-to-volume ratio [6], tunable morphology [7], excellent biocompatibility [8], and superior electrochemical [9] and photoelectrochemical (PEC) activity [10], making them ideal candidates for next-generation diagnostic sensors [11]. Beyond biomedical applications, TiO_2_ NT-based sensors have demonstrated significant potential in gas sensing [12], advancing catalyst design [13] and environmental monitoring [14].

The intrinsic advantages of TiO_2_ NTs stem from their anodized fabrication process [15], which allows precise control over NT dimensions [16], crystallinity [17], and surface functionalization [18]. These properties facilitate enhanced sensitivity, selectivity, and stability in detecting a wide range of biomolecules, from small metabolites (e.g., glucose [19], cholesterol [20], uric acid [21]) to complex biomarkers (e.g., cardiac troponins [22], cancer-derived exosomes [23], and infectious disease agents [24]). Furthermore, their photoactive nature enables the development of PEC biosensors, which offer improved signal-to-noise ratios by decoupling excitation (light) and detection (current) sources [25].

**Scheme 1:**
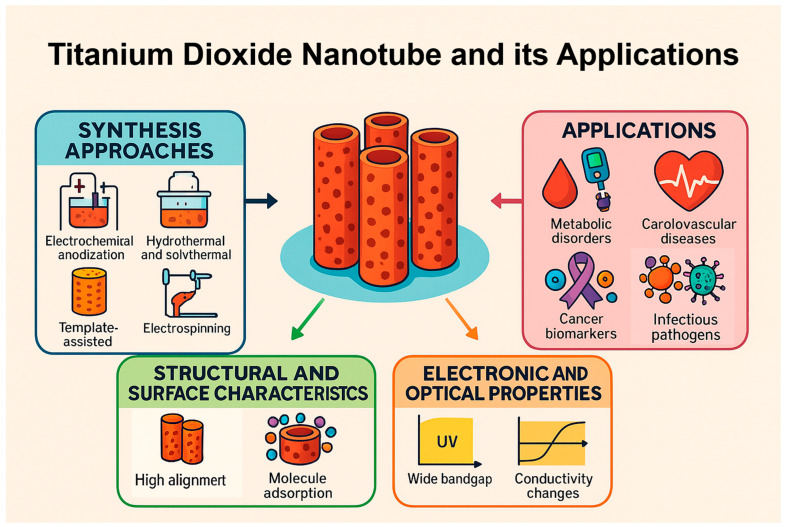
Schematic representation of synthesis procedures, properties, and biomedical applications of TiO_2_ NTs.

This review provides an analysis of the progress and innovation in TiO_2_ NT-based sensing technologies over the past two decades, with a focus on medical diagnostics (Scheme 1). By consolidating foundational research and emerging trends, this review aims to inspire further innovation in TiO_2_ NT-based biosensors, bridging the gap between laboratory-scale breakthroughs and their translation into point-of-care (POC) diagnostic tools for global healthcare applications.

## 2. TiO_2_ Nanotubes: Synthesis and Properties

The unique structural and electronic properties of TiO_2_ NTs make them highly attractive for sensing applications, particularly in medical diagnostics. These properties are largely determined by the synthesis methods employed.

### 2.1. Synthesis Approaches

The synthesis of TiO_2_ NTs is a foundational step in tailoring their physicochemical properties, including morphology, crystallinity, surface area, and defect states [26], for biosensing functionality [27]. This section expands beyond electrochemical anodization, commonly used for its uniformity and scalability, to explore alternative methods like hydrothermal/solvothermal, template-assisted, and electrospinning techniques. These approaches provide unique advantages tailored to diverse sensor design and functional integration needs.

#### 2.1.1. Electrochemical Anodization

Electrochemical anodization [28] remains the most widely adopted method for fabricating highly ordered TiO_2_ NT arrays. It involves the electrochemical oxidation of titanium in fluoride-containing electrolytes under controlled voltage or current regimes [29]. Key parameters such as electrolyte composition, pH, temperature, and anodization duration critically influence tube length, diameter, wall thickness, and crystalline phase [30]. Despite its reproducibility and alignment with device-scale integration, anodization typically necessitates post-annealing [31] to convert amorphous TiO_2_ into bioactive anatase or rutile phases [32].

#### 2.1.2. Hydrothermal and Solvothermal Synthesis

Hydrothermal and solvothermal routes utilize autoclave-based reaction environments [33] to promote the crystal growth of nanotubular TiO_2_ under moderate temperatures [34]. These methods allow for the direct synthesis of anatase-phase nanotubes from Ti precursors in alkaline media [35]. The process involves the dissolution of TiO_2_ and recrystallisation into nanotubular titanate structures, which can be protonated and converted into TiO_2_ anatase nanotubes by washing with acid [36]. Additionally, the solvent system tunes the surface chemistry for selective molecular interactions, critical for biosensing specificity [37]. Additionally, low-temperature processing offers an alternative for substrate-compatible integration [38].

#### 2.1.3. Template-Assisted Synthesis

Template-assisted methods offer a top-down approach wherein TiO_2_ is deposited into preformed nanoporous templates such as anodic aluminum oxide (AAO) [39], polycarbonate membranes [40], or track-etched films [41]. Following deposition, the template is chemically removed to yield free-standing TiO_2_ nanotubes. This method provides exceptional control over geometry and alignment, particularly valuable for directional sensing, optoelectronic coupling, and constructing arrays with multiplexed detection capabilities.

#### 2.1.4. Electrospinning for Hybrid and Composite Nanotubes

Electrospinning has emerged as a versatile technique to fabricate one-dimensional TiO_2_-based nanostructures [42], particularly hybrid or composite nanotubes embedded with secondary phases. Precursor solutions of titanium alkoxides mixed with polymers are electrospun into nanofibers and then calcined to remove organics, resulting in hollow or solid nanotubes depending on the protocol [43]. This approach enables the incorporation of multifunctional sensing domains, essential for next-generation electrochemical or optical biosensors.

For sensing-specific applications, tailoring the structural parameters is essential. Shorter NTs with larger inner diameters are preferred for rapid analyte diffusion, while longer arrays provide a greater number of adsorption sites, improving sensitivity [44].

### 2.2. Structural and Surface Characteristics

The structural integrity and high degree of vertical alignment of TiO_2_ NTs [45] facilitate directional charge transport along the length of the tube, reducing electron scattering and recombination, thereby maintaining high signal-to-noise ratios in electrochemical and PEC sensing platforms. Furthermore, the inner and outer surfaces of the NTs provide dual-active interfaces for molecular adsorption, which enhances the sensor’s interaction with the analyte [46].

Surface properties play a pivotal role in determining sensing performance. The abundance of surface hydroxyl groups [47] and oxygen vacancies [48] on TiO_2_ NTs can be exploited to enhance adsorption affinity and catalytic activity. These defect sites also contribute to the modulation of the local electronic environment, thereby influencing the sensitivity and selectivity of TiO_2_ NT-based sensors.

### 2.3. Electronic and Optical Properties in Sensing Context

TiO_2_ NTs possess a wide bandgap, which restricts their native optical response to the ultraviolet region. However, in sensing applications, this limitation can be overcome by modifying TiO_2_ NTs through doping, surface sensitization with semiconducting nanoparticles (e.g., CdS [49]), or plasmonic nanoparticle decoration (e.g., Au [50]). These strategies effectively extend the photoresponse into the visible region, enabling sunlight-driven PEC sensing and reducing the requirement for high-energy excitation sources.

The semiconducting nature of TiO_2_ enables it to function as a transducer material in resistive [51], capacitive [52] sensor platforms. Analyte binding or redox interaction at the surface alters the local charge density or interfacial potential, resulting in measurable changes in conductivity or photocurrent. Furthermore, TiO_2_ NTs exhibit excellent electron mobility along their axial direction, which facilitates rapid signal transduction and contributes to the high temporal resolution of the sensors [53].

### 2.4. Functional Modifications for Enhanced Sensitivity and Selectivity

The efficacy of TiO_2_NT-based biosensors is heavily influenced by their surface functionalization, which determines their sensitivity, selectivity, and stability [54]. Different functionalization strategies have been developed to optimize performance for specific diagnostic applications. These approaches can be systematically classified into five major categories: enzyme-modified sensors, enzyme-free sensors, antibody/antigen-based immunosensors, aptamer-modified sensors, and molecularly imprinted polymer (MIP) sensors. Each method offers unique advantages and limitations, making them suitable for different biomedical sensing scenarios.

#### 2.4.1. Enzyme-Modified Sensors

Enzyme-modified TiO_2_ NT biosensors utilize biological recognition elements, such as glucose oxidase (GOx) [55], to achieve high specificity. These sensors operate on the principle of redox catalysis, where the enzyme converts the target analyte (e.g., glucose) into an electroactive product (e.g., H_2_O_2_), generating a measurable current or PEC signal. The TiO_2_ NT substrate provides a large surface area for enzyme immobilization, while metal nanoparticles (e.g., Pt) enhance electron transfer efficiency [56]. However, limitations include enzyme instability under varying pH/temperature conditions and interference from electroactive species like ascorbic acid.

#### 2.4.2. Enzyme-Free (Electrocatalytic) Sensors

Enzyme-free sensors rely on nanomaterial-modified TiO_2_ NTs [57,58,59], for direct electrocatalytic oxidation or reduction of target molecules (e.g., glucose). Unlike enzymatic systems, these sensors avoid biological instability by using inorganic catalysts that facilitate analyte-specific redox reactions at lower overpotentials [60]. The detection mechanism is based on direct charge transfer between the analyte and the modified TiO_2_ NT surface, often enhanced by conductive polymers [61]. These sensors offer long-term stability but may require additional surface modifications to improve selectivity.

#### 2.4.3. Antibody-/Antigen-Based Immunosensors

Antibody-functionalized TiO_2_ NT biosensors operate through binding-induced signal modulation, where immunorecognition events (e.g., antibody–antigen binding) produce measurable changes in impedance, fluorescence, or electrochemical signals [62]. These sensors are highly specific, making them ideal for detecting protein biomarkers such as prostate-specific antigen (PSA) [50] or cardiac troponin I [22]. Typically, antibodies are immobilized on TiO_2_ NTs via gold nanoparticles (AuNPs) or chitosan, and detection is often amplified using sandwich-type assays with labeled secondary antibodies. While these systems offer exceptional specificity, challenges include antibody denaturation and complex conjugation chemistry.

#### 2.4.4. Aptamer-Modified Sensors

Aptamer-based TiO_2_ NT biosensors employ synthetic oligonucleotides as recognition elements, which undergo conformational changes upon target binding, leading to measurable signal variations [63]. These sensors combine the specificity of biological recognition with the stability of synthetic DNA/RNA, making them suitable for detecting small molecules, proteins, or even whole cells. Detection mechanisms include electrochemiluminescence (ECL) [64], or current modulation. Aptamers offer advantages such as thermal stability and reusability but may degrade in nuclease-rich biological environments.

#### 2.4.5. Molecularly Imprinted Polymer (MIP) Sensors

MIP-modified TiO_2_ NT sensors use synthetic polymer cavities designed to mimic natural antibody binding sites, enabling selective recognition of target molecules [65]. The detection mechanism often involves analyte rebinding-induced signal changes, such as photocurrent suppression in PEC sensors or impedance shifts in electrochemical setups [66]. MIPs are highly stable and cost-effective, making them suitable for harsh operational conditions. While MIPs avoid biological instability, challenges include template leakage and inconsistent binding site distribution.

The choice of functionalization method for TiO_2_ NTs depends on the target analyte and intended application. While enzyme-modified sensors provide high specificity, enzyme-free alternatives offer greater stability. Immunosensors and aptamer-based designs excel in protein and pathogen detection, whereas MIP sensors combine robustness with synthetic versatility. By systematically optimizing these approaches, TiO_2_ NT-based biosensors can be further refined for clinical and POC applications.

## 3. Applications of TiO_2_ Nanotubes in Medical Diagnostics

**Scheme 2:**
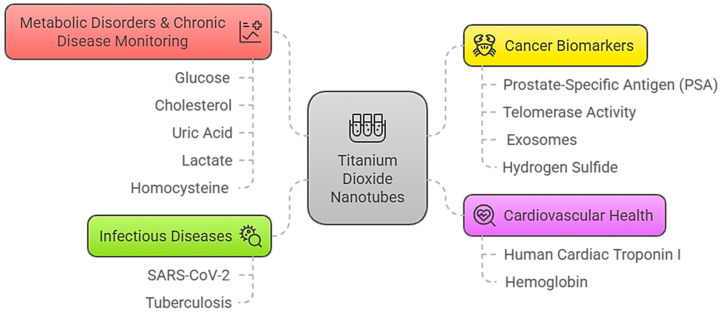
Multifaceted applications of TiO_2_ NTs in medical diagnostics.

TiO_2_ NTs offer exceptional properties for medical diagnostics, including high surface area, electrochemical stability, and biocompatibility. This section highlights their applications in detecting metabolic disorders, cardiovascular diseases, cancer biomarkers, and infectious pathogens (Scheme 2).

### 3.1. Metabolic Disorders and Chronic Disease Monitoring

Accurate measurement of metabolic biomarkers is vital for managing chronic diseases. This section examines TiO_2_ NT sensors designed to detect glucose, cholesterol, uric acid (UA), lactate, and homocysteine—each of which informs on diabetes, cardiovascular risk, gout/renal health, tissue hypoxia/sepsis, and vascular/neurodegenerative risk. The unique demands of these analytes have motivated tailored TiO_2_ NT morphologies and surface treatments to enhance sensitivity, selectivity, and real-time response.

#### 3.1.1. Glucose

**Figure 1:**
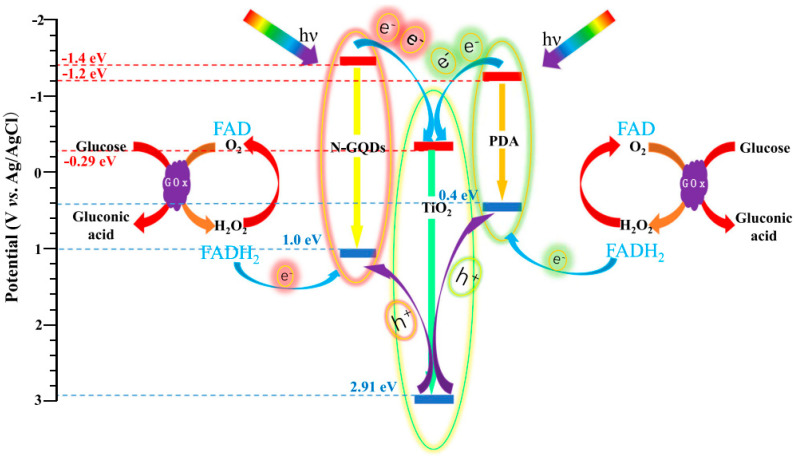
The sensing mechanism of the TiO_2_ NT/PDA/N-GQD/GOx dual-electron-acceptor biosensor (Reproduced with permission [68]).

In the last two decades, a large number of research groups have focused on the detection of glucose due to its critical role in medical diagnostics, especially in the monitoring and management of diabetes mellitus. Accurate and rapid glucose detection is essential for preventing complications associated with abnormal blood sugar levels. TiO_2_ NT-based platforms have been widely explored for glucose sensing due to their high surface area, excellent electron transport properties, and compatibility with diverse surface modifications. Kang et al. [19] developed a glucose biosensor using a Pt–Au nanoparticle-decorated TiOx NT array. Cyclic voltammetry (CV) tests revealed that the Pt–Au/TiOx NT electrode exhibited significantly higher catalytic activity toward H_2_O_2_ oxidation compared to bare or single-metal-modified electrodes. By immobilizing GOx on the modified electrode, the researchers fabricated a glucose biosensor that demonstrated a detection limit of 0.1 mM. The biosensor also showed strong selectivity against common interferents, such as ascorbic acid and UA, and maintained stability for over 25 days, indicating its potential for biomedical sensing applications. In contrast, Li et al. [67] developed a highly sensitive non-enzymatic glucose biosensor using nickel-copper (Ni–Cu) nanoparticle-modified TiO_2_NT arrays prepared via a potential step method. The Ni–Cu/TiO_2_NT electrode exhibited superior electrocatalytic activity for glucose oxidation in alkaline media compared to monometallic Ni or Cu counterparts, achieving a low detection limit of 5 μM. The sensor also demonstrated excellent selectivity against common interferents like ascorbic acid and UA, along with strong long-term stability and reproducibility. Yang et al. [68] introduced an ultrasensitive PEC biosensor for glucose detection by modifying TiO_2_ NTs with polydopamine (PDA) and amino-functionalized graphene quantum dots (N-GQDs). The dual-electron-acceptor structure, formed through electropolymerization of PDA and microwave-assisted loading of N-GQDs, significantly enhanced photoelectric response and electron-hole separation efficiency (Figure 1). The biosensor demonstrated exceptional performance, including a low detection limit of 0.015 mM. More recently, Kumar and Sinha [69] fabricated a highly sensitive non-enzymatic glucose biosensor using silver nanoparticles and Cu_2_O deposited on TiO_2_ NT arrays. The TiO_2_ NTs were synthesized via anodization on a Ti6Al4V titanium alloy substrate, followed by sequential deposition of Cu_2_O through wet chemical bath methods and silver via electro-deposition. The incorporation of Cu_2_O and Ag significantly enhanced the electroactive surface area, facilitating rapid electron transfer and resulting in excellent electrochemical performance. Electrochemical studies revealed a low detection limit (36 µM) and reliable glucose detection in human serum, demonstrating outstanding selectivity, reproducibility, and stability. These studies collectively demonstrate that both enzymatic and enzyme-free TiO_2_ NT-based biosensors can be finely tuned through nanomaterial engineering to achieve high sensitivity, selectivity, and practical applicability.

#### 3.1.2. Cholesterol

Cholesterol detection is vital for diagnosing and managing cardiovascular diseases, monitoring metabolic health, and preventing conditions like atherosclerosis and stroke. Khaliq et al. [70] demonstrated that Cu_2_O nanoparticle decoration of TiO_2_ NTs—achieved via sonication-assisted chemical bath deposition—yields a five-fold enhancement in sensitivity (6 034.04 µA mM^−1^ cm^−2^) over pristine TiO_2_ NTs, and a detection limit as low as 0.05 µM; these gains were ascribed to the expanded electroactive surface area and the intimate Cu_2_O–TiO_2_ heterojunction that accelerates charge transport, while selectivity against glucose and ascorbic acid and concordance with commercial enzymatic assays in human serum emphasized the platform’s practical viability. Building on this foundation, Kumar and Sinha [20] reported that anodized TiO_2_ NTs bearing uniformly dispersed Cu_2_O nanoparticles deliver an even higher sensitivity of 10 981.25 µA mM^−1^ cm^−2^ and a detection threshold of 0.042 mM. CV and electrochemical impedance spectroscopy (EIS) revealed that the Cu_2_O@TiO_2_NTs nanocomposite markedly lowers charge-transfer resistance, while interference studies (ascorbic acid and UA) and 25-day stability tests validated its robustness for real-time serum cholesterol analysis—although long-term durability beyond one month and an in-depth mechanistic elucidation of the Cu_2_O–cholesterol redox pathway remained to be addressed. Extending this concept further, the same authors [71] subsequently engineered a bimetal-oxide/metal hybrid by co-depositing Ag nanoparticles and Cu_2_O onto TiO_2_ NTs fabricated from Ti6Al4V alloy: the resulting Ag–Cu_2_O@TiO_2_NTs architecture exhibited a peak sensitivity of 12,140.06 µA mM^−1^ cm^−2^, a 0.057 mM detection limit, and superior electron-transfer kinetics as evidenced by EIS, along with excellent anti-interference capability, 21-day operational stability, and accurate serum cholesterol quantification. These studies illustrate a clear, iterative progression—Cu_2_O decoration, optimized nanoparticle dispersion, and synergistic Ag–Cu_2_O coupling—that systematically enhances electroactive surface area, reduces charge-transfer resistance, and fortifies selectivity and stability, thereby charting a path toward clinically viable, non-enzymatic TiO_2_ NT-based cholesterol biosensors.

#### 3.1.3. Uric Acid

**Figure 2:**
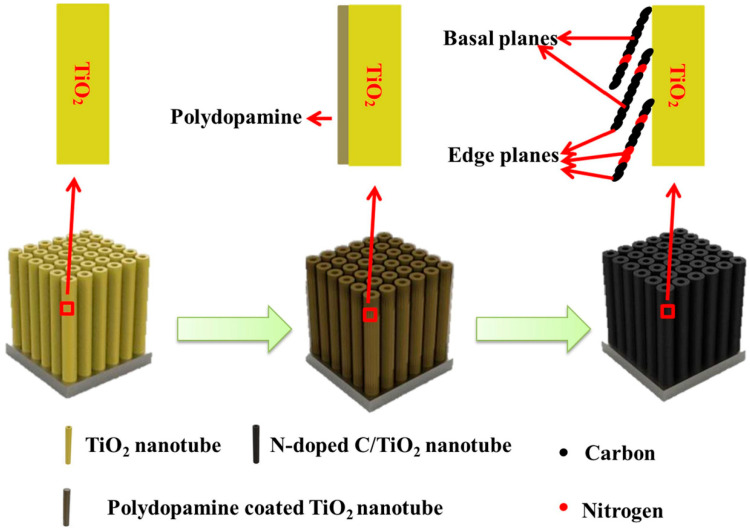
Schematic representation of the fabrication process for N-doped carbon-coated TiO_2_ NT arrays (Reproduced with permission [72]).

Uric acid (UA) detection plays a critical role in the diagnosis and management of metabolic disorders such as gout, hyperuricemia, hypertension, and renal dysfunction. Lee et al. [21] established a foundation for TiO_2_ NT-based biosensing by fabricating well-ordered NT arrays through anodic oxidation of titanium in an optimized ethylene glycol-based electrolyte system. These arrays demonstrated excellent electrochemical responsiveness, as evidenced by high linearity in the amperometric detection of UA across concentrations ranging from 2 to 14 mg/dL, with sensitivities of 23.3 (μA·cm^−2^)·(mg/dL)^−1^. Building upon this platform, Wang et al. [72] introduced a catalyst-free strategy that overcame the limitations associated with residual catalytic impurities, which often confound electrochemical signal interpretation (Figure 2). By employing a template carbonization process of polydopamine-coated TiO_2_ NT arrays, they successfully synthesized N-doped carbon-coated TiO_2_ NT arrays with a uniform morphology, high electrochemical surface area, and superior electron transfer characteristics. These electrodes achieved heterogeneous charge transfer rate constants exceeding 0.5 cm/s for the [Fe(CN)_6_]^3−^/^4−^ redox couple and facilitated the simultaneous, selective quantification of UA over broad dynamic ranges, with a detection limit of 0.11 μM. More recently, Ma et al. [49] designed a sophisticated PEC biosensing platform by engineering a CdS/Au/TiO_2_ Z-scheme heterojunction on Ti foil via the SILAR method, obviating the need for conductive substrates or binders. This heterostructure enabled efficient separation and migration of photoexcited charge carriers, thereby significantly enhancing photocurrent response under visible light illumination. The integration of MIPs as recognition elements further endowed the system with high selectivity, enabling real-time, non-invasive detection of UA in saliva with an ultralow detection limit of 5.07 nM and a broad linear range from 0.01 to 50 μM. Importantly, the analytical performance of this portable PEC sensor showed no statistically significant deviation when benchmarked against conventional high-performance liquid chromatography (HPLC), emphasizing its potential for deployment in POC diagnostics.

#### 3.1.4. Lactate

Given the clinical significance of lactate as a critical biomarker for conditions such as sepsis, tissue hypoxia, and metabolic disorders, its precise and timely quantification is essential for effective patient management and diagnosis. In response to this necessity, Cheng et al. [73] developed a robust solid-state ionic biosensor for lactate quantification, employing a composite membrane exhibiting a graphene-like architecture. This engineered film was synthesized via copolymerization, integrating TiO_2_ NTs, polyaniline (PANI), the ionic liquid 1-ethyl-3-vinylimidazolium chloride (EVIMC), and chloroauric acid (HAuCl_4_), resulting in a highly ordered nanostructure with superior electrocatalytic attributes. The immobilization of lactate dehydrogenase (LDH), supplemented with isocitrate dehydrogenase (NAD^+^), was facilitated through electrostatic interactions with the membrane, enabling effective catalytic conversion of lactate. The modified electrode, designated as LDH/Au-EVIMC-TiO_2_NTs-PANI/ITO, demonstrated an approximately twofold enhancement in electrochemical response compared to its LDH/TiO_2_NTs/PANI/ITO counterpart. This platform exhibited dual linear dynamic ranges extending from 5.5 × 10^−7^ M to 5.55 × 10^−6^ M and from 5.55 × 10^−6^ M to 3.33 × 10^−3^ M, achieving an exceptional limit of detection (LOD) of 1.65 × 10^−7^ M. Notably, interference assays involving coexisting biological species such as UA and hemoglobin confirmed the biosensor’s high selectivity toward lactate without signal overlap. The system also demonstrated excellent analytical performance in complex biological matrices, delivering recovery rates between 96.7% and 105.8% in real blood sample analysis, thereby affirming its reliability and stability for clinical applications.

#### 3.1.5. Homocysteine

Hung et al. [74] addressed the need for effective homocysteine detection, as it is a sulfur-containing amino acid linked to various health conditions. Their study focused on developing homocysteine biosensors entirely constructed on anodized TiO_2_ NTs, utilizing a two-electrode system. They employed a D-amino acid oxidase enzyme immobilized on TiO_2_ NTs as the working electrode and a bare TiO_2_ NT as the reference electrode. Electrochemical analysis using CV revealed a reversible reaction with a sensitivity of 53.4 nA/μM, a linearity correlation coefficient of 0.998 within a detection range of 0 to 70 μM, and a LOD of approximately 1.5 μM. The researchers concluded that using TiO_2_ NTs for both electrodes in the biosensor system offered advantages such as good enzyme adhesion, significant reaction currents, high sensitivity, and the creation of a reversible electrochemical system, potentially simplifying fabrication for chip-based applications.

### 3.2. Cardiovascular Health

#### 3.2.1. Human Cardiac Troponin I

**Figure 3:**
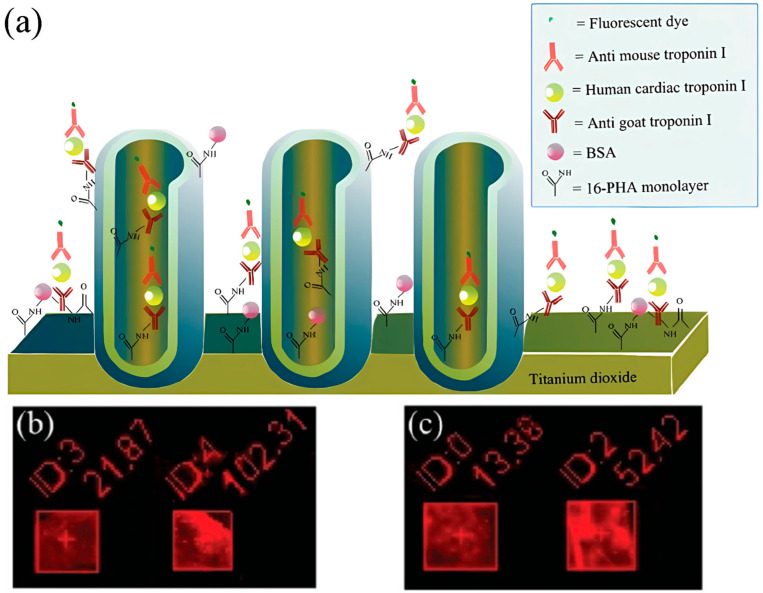
(**a**) Schematic representation of the sandwich-type immunoassay designed for the detection of human cardiac troponin I (cTnI); (**b**) fluorescence micrographs depicting arrays of 900 nm-long transparent TiO_2_ NTs on Kapton substrate, exposed to cTnI concentrations of 0 µg/mL (control, left) and 10 µg/mL (right); (**c**) fluorescence micrographs showing functionalized arrays of 5 µm-long transparent TiO_2_ NTs on Kapton, subjected to cTnI concentrations of 0 µg/mL (control, left) and 0.1 µg/mL (right) (Reproduced with permission [22]).

The sensitive detection of cardiac troponin I, a critical biomarker for myocardial infarction, necessitates the development of high-performance biosensing platforms. However, the integration of self-organized TiO_2_ NT arrays into flexible, transparent, and disposable biosensors has been impeded by the high-temperature requirements (≥300 °C) for depositing titanium films with suitable morphology, adhesion, and transparency on polymer substrates. To overcome this, Farsinezhad et al. [22] employed an atomic peening-based film growth technique enabling the fabrication of highly ordered, transparent TiO_2_ NT arrays (up to 5.1 μm) on polyimide (Kapton) at room temperature, eliminating the need for substrate heating, biasing, or ion sources (Figure 3). The resulting arrays exhibited high optical quality, as indicated by specular reflectivity and periodic interferometric fringes used to extract the air–TiO_2_ composite refractive index. A fluorescent immunoassay biosensor utilizing these arrays achieved cardiac troponin I detection down to 0.1 μg ml^−1^.

#### 3.2.2. Hemoglobin

Hemoglobin detection is essential for diagnosing and monitoring anemia, blood disorders, and overall oxygen-carrying capacity. It also serves as a critical biomarker in assessing chronic diseases like diabetes and kidney dysfunction. Gao et al. [75] introduced an innovative PEC sensor for human hemoglobin detection, constructed using TiO_2_ NT arrays modified with cadmium sulfide (CdS) quantum dots and further enhanced through molecular imprinting techniques. The heterojunction formed between CdS and TiO_2_ significantly improved visible-light-induced charge separation and photocurrent generation. A MIP film, synthesized via dopamine polymerization, was developed to impart target-specific binding affinity toward hemoglobin. Upon target recognition, the bound hemoglobin catalyzed the H_2_O_2_-mediated oxidation of 4-chloro-1-naphthol, yielding an insoluble product that inhibited photocurrent flow, thus allowing sensitive transduction. The PEC sensor demonstrated excellent analytical performance with a dynamic response range from 0.01 to 100 ng/mL and an ultralow detection limit of 0.53 pg/mL. Its applicability was validated through successful deployment in human urine sample analysis, indicating its utility for non-invasive clinical diagnostics.

### 3.3. Cancer Biomarkers

#### 3.3.1. Prostate-Specific Antigen (PSA)

**Figure 4:**
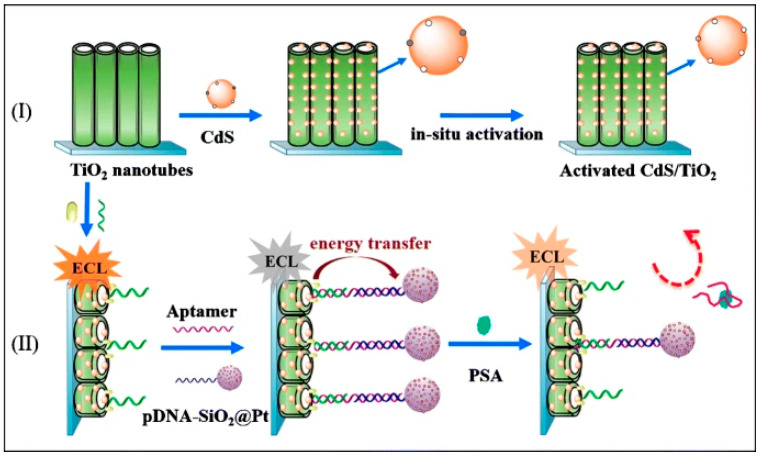
(I) Schematic representation of the construction process for the enhanced cathodic ECL system and (II) ECL system’s plication in PSA detection (Reproduced with permission [64]).

PSA (Prostate-Specific Antigen) detection is essential for early diagnosis, monitoring, and management of prostate cancer and related prostate disorders. In 2018, Kiziltan et al. [50] designed an electrochemical immunosensor for PSA detection by modifying disposable titanium electrodes through anodic oxidation, producing a nanoporous titania surface. These anodized electrodes, optimized through controlled anodization parameters, were further functionalized with gold nanoparticles (AuNPs) to enhance electrical conductivity and chitosan to facilitate biomolecule immobilization. Anti-PSA antibodies were covalently attached to the modified surface to achieve specific antigen recognition. The sensor demonstrated a linear detection range of 0 to 100 ng/mL, with LOD calculated at 7.8 ng/mL. In a subsequent advancement, Dai et al. [64] introduced a highly sensitive cathodic ECL assay for PSA employing TiO_2_ NTs decorated with thioglycolic acid-capped CdS nanocrystals as efficient ECL emitters (Figure 4). Upon activation with a hydrogen peroxide–citric acid system, the ECL intensity of the CdS/TiO_2_ NTs increased up to 265-fold in comparison to unmodified TiO_2_ NTs. The detection system was constructed by hybridizing complementary DNA, PSA-specific aptamers, and probe DNA-functionalized SiO_2_@Pt nanoparticles onto the CdS/TiO_2_ NTs. In the presence of PSA, the aptamer binding caused the release of quencher-labeled DNA probes, producing a significant “off-on” enhancement in the ECL signal. The platform enabled highly sensitive PSA detection with a broad dynamic range from 0.001 to 50 ng/mL and an ultralow LOD of 0.4 pg/mL, demonstrating strong potential for clinical diagnostic applications.

#### 3.3.2. Telomerase Activity

Telomerase activity detection is crucial for early cancer diagnosis, prognosis, and therapeutic targeting, as well as for understanding aging and cellular longevity. Dai et al. [76] developed a portable dual-mode sensor for telomerase detection using a TiO_2_ NT membrane functionalized with a DNA primer (template strand) immobilized through phosphate-Ti(IV) bidentate binding. The sensor employed AuNP-tagged reporter DNA for hybridization with telomerase-extended primers, followed by silver amplification to enhance signal blockage in the nanochannels, achieving an ultra-low detection limit of 0.8 HeLa cells. This system enabled both qualitative naked-eye analysis (via color change from yellow to pink/grey) and quantitative electrochemical detection through ionic current reduction, demonstrating high sensitivity and specificity in clinical urine samples from bladder cancer patients. The immobilized DNA-AuNP complex, combined with catalytic silver deposition, allowed for dual-mode telomerase activity evaluation, offering a rapid, non-invasive diagnostic tool with superior performance compared to conventional methods.

#### 3.3.3. Exosomes

Recent advances in cancer diagnostics have highlighted exosomes as promising biomarkers for early disease detection, yet the reliable identification of tumor-derived exosomes in biological fluids presented significant technical difficulties. To address this challenge, He et al. [23] developed an unlabeled electrochemical biosensing platform employing TiO_2_ NT arrays for the precise quantification of exosomal particles in complex matrices. The system employed the unique electrochemical characteristics of TiO_2_ NT arrays, particularly their selective affinity for phosphate moieties present on exosomal membranes, which induced measurable perturbations in ionic and electronic transport through the nanotubular architecture. These charge transfer modifications generated quantifiable signal variations, permitting exosome detection across a broad dynamic range (5 × 10^1^ to 1 × 10^7^ particles/μL), with exceptional sensitivity demonstrated by limits of detection at 12.7 particles/μL for hepatocellular carcinoma-derived exosomes and 12.6 particles/μL for colon cancer-derived vesicles. Clinical validation studies confirmed the platform’s diagnostic capability, successfully discriminating extracellular vesicle signatures in serum samples from hepatocellular carcinoma patients (*n* = 20), colon cancer patients (*n* = 20), and healthy controls (*n* = 20), with statistically significant differentiation (*p* < 0.0001).

#### 3.3.4. Hydrogen Sulfide

Hydrogen sulfide (H_2_S) detection is vital in cancer research as it plays a key role in tumor progression, metastasis, and the regulation of cellular pathways involved in cancer development. A novel PEC sensing platform was developed for the sensitive detection of H_2_S through an innovative semiconductor heterostructure design by Ding et al. [77]. They fabricated this system by covalently anchoring CdS nanoparticles onto TiO_2_ NT arrays via a thioglycolic acid surface modification strategy, which introduced thiol functional groups for subsequent cadmium ion immobilization. Upon exposure to either exogenous sources or endogenous H_2_S released from cancer cells, these immobilized cadmium precursors underwent in situ conversion to photoelectrochemically active CdS nanoparticles. The resulting CdS-TiO_2_ heterojunction exhibited enhanced photocurrent generation due to improved charge separation efficiency at the semiconductor interface. Analytical characterization demonstrated excellent sensor performance across a broad dynamic range spanning five orders of magnitude (10–10^6^ nM), with an exceptionally low detection limit of 0.7 nM achieved through optimized interfacial charge transfer kinetics. This platform successfully detected both chemically introduced H_2_S and biologically derived H_2_S from cellular systems, establishing its potential for biomedical applications.

### 3.4. Infectious Diseases

#### 3.4.1. SARS-CoV-2

**Figure 5:**
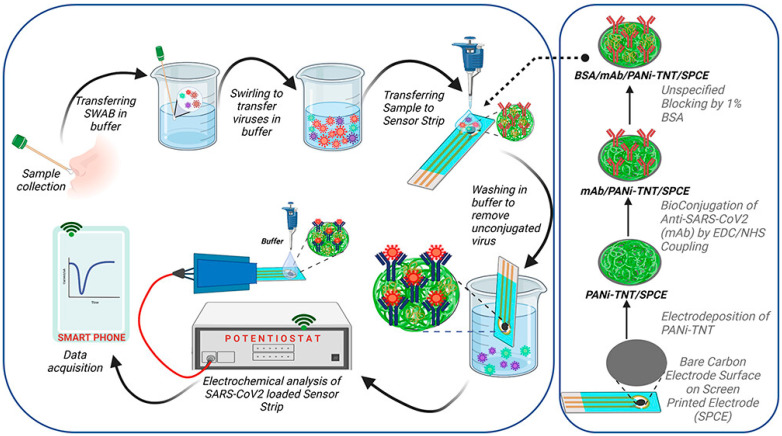
Schematic representation of a polyaniline–titania NT-based electrochemical biosensor strip engineered for the sensitive and selective detection of SARS-CoV-2 through specific antigen–antibody interactions (Reproduced with permission [78]).

The Coronavirus Disease 2019 (COVID-19), attributed to the novel pathogen severe acute respiratory syndrome coronavirus 2 (SARS-CoV-2), rapidly escalated into a global health crisis shortly after its initial emergence, prompting the World Health Organization (WHO) to designate it a pandemic. In response to this urgent need, Vadlamani et al. [24] fabricated a cost-effective yet highly responsive electrochemical biosensor based on Co-modified TiO_2_ NTs, specifically engineered for the rapid detection of the SARS-CoV-2 spike protein receptor-binding domain (S-RBD). The TiO_2_ NT architecture was synthesized via a one-step electrochemical anodization protocol, subsequently functionalized with Co using an incipient wetting technique, and integrated with a potentiostat for electrochemical readout. This sensor platform demonstrated exceptional specificity and sensitivity towards the SARS-CoV-2 S-RBD, achieving reliable detection within approximately 30 s across a concentration spectrum of 14 to 1400 nM. Later on, Alam et al. [78] developed an innovative biosensor strip tailored for the rapid and ultrasensitive electrochemical identification of SARS-CoV-2. This diagnostic platform was fabricated by immobilizing a SARS-CoV-2-specific monoclonal antibody (mAb) onto a screen-printed carbon electrode (SPCE) surface that had been previously modified with a composite of polyaniline and titania NTs, thereby enhancing the electrochemical responsiveness of the sensor interface (Figure 5). The developed biosensing element was integrated into a portable detection system, capable of wireless communication with devices operating on Windows or Android platforms. Upon introduction of the SARS-CoV-2 spike protein, a rapid and highly specific antigen–antibody interaction occurred, which was quantitatively monitored using chronoamperometric techniques in a phosphate-buffered saline medium. Analytical characterization demonstrated that the biosensor exhibited high sensitivity and a linear detection response within the concentration range of 80 to 200 copies per μL, achieving a LOD as low as 25.59 copies/μL. The selectivity of the biosensor was rigorously validated through comparative assays, confirming its ability to distinguish SARS-CoV-2 from other viral pathogens, including human coronavirus strains HCoV-OC43, HCoV-NL63, HCoV-229E, and adenovirus.

#### 3.4.2. Tuberculosis

The electrochemical detection of tuberculosis biomarker vapors using Co-functionalized TiO_2_ NT arrays has been investigated by Smith et al. [79], demonstrating promising potential for non-invasive, POC diagnostics. They synthesized highly ordered TiO_2_ NT arrays via electrochemical anodization and achieved in situ functionalization with cobalt hydroxide (Co(OH)_2_) during the anodization process. This sensor system exhibited enhanced sensitivity and faster response times compared to conventional post-synthesis functionalization techniques, such as wet incipient impregnation, when exposed to TB biomarkers including methyl nicotinate, methyl p-anisate, methyl phenylacetate, and o-phenylanisole at concentrations between 275 and 360 ppm. The sensing mechanism was primarily governed by electron transfer from the biomarkers to Co^2+^/Co redox sites, improving conductivity and selectivity over common breath volatiles like methanol and acetone. In a complementary study, Bhattacharyya et al. [80] employed a similar approach but utilized incipient wetness impregnation for Co(OH)_2_ deposition onto electrochemically anodized TiO_2_ NTs. Their sensor achieved an impressive detection limit of ~0.018 ppm, with methyl p-anisate showing the highest current response (81 μA) and methyl nicotinate exhibiting the fastest reaction kinetics (35 s). The enhanced performance was attributed to the comparable dimensions of the depletion layer and NT wall thickness, facilitating efficient electron transfer. Butler–Volmer modeling and Tafel analysis further confirmed the redox behavior of the biomarkers, aligning with the observed sensor responses.

The prior discussion of TiO_2_ NT-based biosensing platforms is summarized in Table 1.

## 4. Challenges and Future Scope

The remarkable progress in TiO_2_ NT-based sensing technologies for medical diagnostics has been accompanied by several persistent challenges that must be addressed to enable widespread clinical adoption. While these nanostructured platforms demonstrate exceptional sensitivity and versatility in biomarker detection, key limitations in fabrication reproducibility, long-term stability, and device integration continue to hinder their translation from research laboratories to practical POC applications. Overcoming these challenges while capitalizing on emerging technological opportunities will be crucial for realizing the full potential of TiO_2_ NT sensors in modern healthcare.

A primary challenge lies in achieving consistent, large-scale fabrication of TiO_2_ NT arrays with uniform morphological and electronic properties [81]. Although electrochemical anodization provides excellent control over nanotube dimensions at the laboratory scale, minor variations in process parameters such as electrolyte composition [82], applied voltage, and temperature can significantly impact the resulting nanostructure [83,84]. This sensitivity to synthesis conditions creates reproducibility issues that complicate mass production and quality control. Furthermore, conventional high-temperature annealing required for crystallinity poses limitations for flexible or disposable sensor platforms using polymer substrates [85]. Recent advances in low-temperature processing and alternative synthesis methods like plasma electrolytic oxidation show promise for addressing these scalability challenges.

The long-term operational stability of TiO_2_ NT sensors in complex biological environments presents another critical hurdle [86]. While the material itself exhibits excellent chemical inertness [87], performance degradation can occur through multiple mechanisms including surface fouling by proteins, photocorrosion in PEC systems, and instability of functional components like enzymes or noble metal nanoparticles [88]. For instance, even highly sensitive glucose biosensors employing GOx on TiO_2_ NTs typically show reduced activity after several weeks due to enzyme denaturation [89]. Developing robust surface modification strategies and protective coatings will be essential to enhance sensor longevity for continuous monitoring applications.

Selectivity remains an ongoing challenge, particularly for electrochemical detection of structurally similar analytes in multicomponent biological fluids [90]. While molecular imprinting and biorecognition elements (antibodies, aptamers) have improved specificity, interference from compounds like ascorbic acid and uric acid continues to complicate measurements [20]. Emerging approaches combining advanced material engineering with machine learning-assisted signal processing may provide solutions by enabling multiplexed detection and pattern recognition of complex biomarker signatures.

The integration of TiO_2_ NT sensors into portable, user-friendly devices represents a significant technological barrier [91]. Most current prototypes rely on benchtop instrumentation for readout, limiting their utility in resource-constrained or POC settings. Miniaturization challenges include maintaining sensitivity while reducing device footprint, developing low-power detection schemes, and implementing reliable sample handling systems [92]. Recent work on paper-based TiO_2_ NT sensor [93] technologies demonstrate progress toward truly portable diagnostic platforms.

In addition to technical considerations, the clinical translation of TiO_2_ NT-based sensors requires careful attention to regulatory, validation, and interoperability frameworks. Successful commercialization hinges on adherence to regulatory standards such as ISO 13485 [94] for medical device quality systems and the FDA [95]/EMA [96] guidelines for in vitro diagnostics. Rigorous clinical validation protocols, including blinded cohort studies [97], specificity/sensitivity benchmarks, and reproducibility metrics under real-world conditions, are essential for demonstrating clinical utility. Furthermore, integration into existing diagnostic workflows—such as compatibility with lateral flow formats [98], smartphone readouts, or centralized laboratory systems—must be strategically addressed to ensure seamless adoption. Collaborations among academic researchers, clinical laboratories, and industry partners will be crucial in navigating these translational pathways and achieving regulatory and market readiness.

**Scheme 3:**
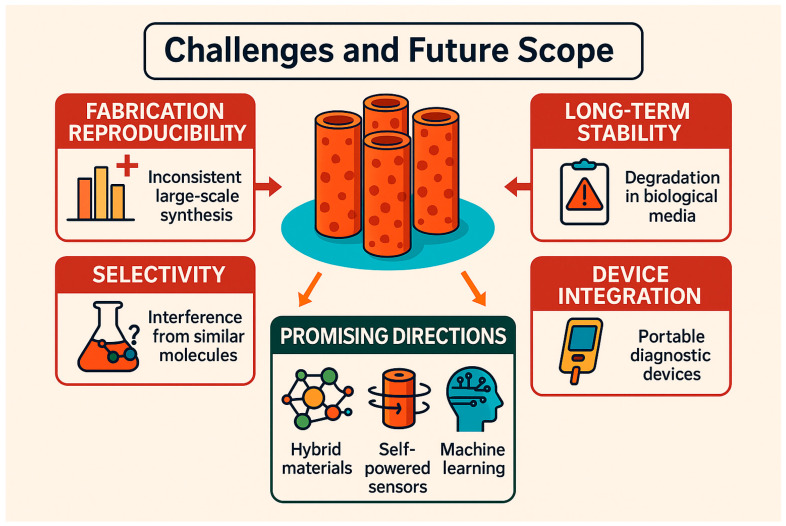
Schematic representation of challenges and future scope of TiO_2_ NTs in biomedical applications.

Looking ahead, several promising directions could overcome these limitations while opening new opportunities. Hybrid material systems combining TiO_2_ NTs with two-dimensional materials (e.g., graphene [68]) or conductive polymers (e.g., PANI [73]) may enhance both sensitivity and stability through synergistic effects. The development of self-powered sensors [99] exploiting the piezoelectric or triboelectric properties of TiO_2_ could enable autonomous operation for implantable or wearable applications [100]. Artificial intelligence and machine learning approaches offer powerful tools for analyzing complex sensor responses and compensating for environmental variability (Scheme 3).

## 5. Conclusions

Over the past two decades, TiO_2_ NTs have emerged as a transformative platform for medical diagnostics, offering unparalleled advantages in sensitivity, versatility, and functional adaptability. This review has highlighted the remarkable progress in TiO_2_ NT-based sensing technologies, from their controlled synthesis and tunable properties to their diverse applications in detecting metabolic biomarkers, cardiovascular indicators, cancer signatures, and infectious disease agents. The unique structural and electronic characteristics of TiO_2_ NTs, combined with innovative surface modifications and hybrid material integrations, have enabled the development of biosensors with exceptional performance metrics, including ultra-low detection limits, high selectivity, and rapid response times. However, the transition from laboratory prototypes to clinically viable devices necessitates overcoming persistent challenges in fabrication reproducibility, long-term stability, and scalable integration into POC systems. Future advancements in nanomaterial engineering, artificial intelligence-driven signal processing, and miniaturized device design hold immense promise for addressing these limitations. As interdisciplinary collaborations continue to bridge the gap between fundamental research and practical implementation, TiO_2_ NT-based sensors are poised to revolutionize personalized medicine, enabling real-time, non-invasive, and cost-effective diagnostic solutions for global healthcare challenges. The next decade will likely witness the maturation of these technologies, cementing their role as indispensable tools in modern medical diagnostics.

## Figures and Tables

**Table 1:** Key parameters of TiO_2_ NT-based biosensing platforms.

Material Composition	Sensor Type	Target	LOD	Sensitivity	Response Time	Reference
Pt–Au/TiOx NT	Electrochemical	Glucose	0.1 mM	0.08366 µA mM^−1^	3 s	Kang et al. [19]
Ni–Cu/TiO_2_ NT	Electrochemical	Glucose	5 μM	1590.9 μA mM^−1^ cm^−2^	5 s	Li et al. [67]
TiO_2_ NTs/PDA/N-GQD	Photoelectrochemical	Glucose	0.015 mM	13.6 μA mM^−1^ cm^−2^	1 s	Yang et al. [68]
Ag-Cu_2_O@TiO_2_ NT	Electrochemical	Glucose	36 µM	1010.20 μA mM^−1^ cm^−2^	2 s	Kumar and Sinha [69]
Cu_2_O/TiO_2_ NT	Electrochemical	Cholesterol	0.05 µM	6034.04 μAmM^−1^ cm^−2^	3 s	Khaliq et al. [70]
Cu_2_O/TiO_2_ NT	Electrochemical	Cholesterol	0.042 mM	10981.25 μAmM^−1^ cm^−2^	3 s	Kumar and Sinha [20]
Ag-Cu_2_O@ TiO_2_ NT	Electrochemical	Cholesterol	0.057 mM	12140.06 μAmM^−1^ cm^−2^	3 s	Kumar and Sinha [71]
Ti/ATiO_2_ NTs	Electrochemical	Uric Acid	30 μM	394 μAmM^−1^ cm^−2^	NA	Lee et al. [21]
N-doped C/TiO_2_ NT	Electrochemical	Uric Acid	0.11 μM	NA	NA	Wang et al. [72]
CdS/Au/TiO_2_-NTs Z-Scheme	Photoelectrochemical	Uric Acid	5.07 nM	50.67 μAμM^−1^ cm^−2^	NA	Ma et al. [49]
Au-EVIMC-TiO_2_ NTs-PANI	Electrochemical	Lactate	1.65 × 10^−7^ M	NA	8 s	Cheng et al. [73]
TiO_2_ NT immobilized with D-amino acid oxidase	Electrochemical	Homocysteine	1.5 μM	53.4 nA/μM	NA	Hung et al. [74]
Transparent TiO_2_ NT arrays	Fluorescent immunoassay	Cardiac Troponin I	0.1 μg/mL	NA	NA	Farsinezhad et al. [22]
MIP/CdS/TiO_2_ NT	Photoelectrochemical	Hemoglobin	0.53 pg/mL	NA	NA	Gao et al. [75]
AuNPs/Chitosan-modified TiO_2_ NT	Electrochemical	PSA	7.8 ng/mL	NA	NA	Kiziltan et al. [50]
CdS/TiO_2_ NT	Electrochemiluminescent	PSA	0.4 pg/mL	NA	NA	Dai et al. [64]
TiO_2_ NT membrane	Colorimetric/Electrochemical	Telomerase	0.8 HeLa cells	NA	NA	Dai et al. [76]
TiO_2_ NT array	Electrochemical	Exosome	12.6 particles/μL	NA	NA	He et al. [23]
TiO_2_ NT/CdS nanoparticles	Photoelectrochemical	H_2_S	0.7 nM	NA	NA	Ding et al. [77]
Co-functionalized TiO_2_ NTs	Electrochemical	SARS-CoV-2	0.7 nM	NA	30 s	Vadlamani et al. [24]
PANi-TiO_2_ NT	Electrochemical	SARS-CoV-2	25.59 copies/μL	NA	30 s	Alam et al. [78]
Co(OH)_2_-functionalized TiO_2_ NTs	Electrochemical	Methyl nicotinate, methyl p-anisate, methyl phenylacetate, o-phenylanisole	~0.018 ppm	NA	35 s	Bhattacharyya et al. [80]

TiO_2_ NTs have demonstrated remarkable potential in medical diagnostics due to their superior physicochemical properties, enabling sensitive and selective detection of diverse biomarkers. For metabolic disorders, enzymatic and non-enzymatic TiO_2_ NT-based sensors achieved low detection limits through tailored modifications like bimetallic nanoparticles (Pt–Au, Ni–Cu) and heterojunctions (CdS/Au/TiO_2_). In cardiovascular diagnostics, transparent TiO_2_ NT arrays on flexible substrates detected cardiac troponin I, while PEC sensors with MIPs quantified hemoglobin. For cancer biomarkers, TiO_2_ NTs enabled ultrasensitive detection of PSA, telomerase, and exosomes, leveraging phosphate affinity and signal amplification strategies. In infectious disease diagnostics, Co-functionalized TiO_2_ NTs detected SARS-CoV-2 spike protein and tuberculosis biomarkers with rapid response times. These advancements collectively emphasize the versatility of TiO_2_ nanotubes as a foundational platform for next-generation biomedical sensing technologies.

## Data Availability

No new data were created or analyzed in this study.
